# Protective Effects of Propolis Supplementation on Aflatoxin B1‐Induced Oxidative Stress, Antioxidant Status, Intestinal Barrier Damage, and Gut Microbiota in Rats

**DOI:** 10.1002/mnfr.70052

**Published:** 2025-03-30

**Authors:** Sevtap Kabalı, Mehtap Ünlü Söğüt, Neslihan Öner, Ayça Kara

**Affiliations:** ^1^ Department of Nutrition and Dietetics Faculty of Health Sciences Ondokuz Mayıs University Samsun Türkiye; ^2^ Department of Nutrition and Dietetics Faculty of Health Sciences Erciyes University Kayseri Türkiye; ^3^ Genome and Stem Cell Center (GENKOK) Erciyes University Kayseri Türkiye

**Keywords:** Aflatoxin B1, gut microbiota, intestinal barrier, oxidative stress, propolis

## Abstract

Aflatoxin B1 (AFB1) is common in the diets of humans and animals and often leads to adverse health effects. Propolis, with its strong antioxidant activity, can reduce oxidative stress and modulate gut microbiota composition. However, the underlying mechanism by which propolis alleviates AFB1‐induced intestinal barrier damage remains unclear. This study was designed to investigate the protective effects of oral propolis supplementation in AFB1‐exposed rats. Thirty‐two male Sprague‐Dawley rats were divided into four groups: control, AFB1, propolis, and AFB1+propolis. After 4 weeks, serum oxidative stress markers were examined, and gut microbiota was analyzed by 16S rRNA sequencing. Intestinal sections were processed by Hematoxylin & Eosin staining, and the expression level of tight junction proteins was assessed by immunostaining. Propolis supplementation in AFB1‐exposed rats tended to decrease oxidative stress, and it also restructured the gut microbiota by preventing a decrease in the relative abundances of *Lactobacillus*, *Roseburia*, and *Phascolarctobacterium*. Propolis restored intestinal permeability impaired by AFB1 by ameliorating intestinal morphological damage and increasing the expression levels of tight junction proteins. Propolis supplementation may contribute to the modulation of gut microbiota by alleviating oxidative stress and improving intestinal barrier damage in AFB1‐exposed rats.

## Graphical abstract

In rats exposed to aflatoxin B1 (AFB1), propolis supplementation may alleviate oxidative stress by exhibiting antioxidant effects and restore intestinal permeability impaired by AFB1 by ameliorating intestinal barrier damage. Furthermore, propolis supplementation may contribute to the modulation of the gut microbiota by preventing a decrease in the relative abundance of several beneficial bacteria.

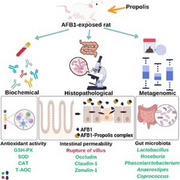

**Abbreviations**
AFB1: Aflatoxin B1
CAT: catalase
GSH: glutathione
GSH‐PX: glutathione peroxidase
H & E: Hematoxylin & Eosin
LDA: linear discriminant analysis
MDA: malondialdehyde
OTU: operational taxonomic unit
ppb: parts per billion
SOD: superoxide dismutase
T‐AOC: total antioxidant capacity

## 1 Introduction

Aflatoxins are mycotoxins produced by the *Aspergillus* *spp*., especially *A. flavus*, *A. parasiticus*, and *A. nomius* species, and naturally occur in food and feed products as they present the most accommodating temperatures and levels of humidity [1]. This toxin is found in grains, legumes, nuts, and spices that are improperly harvested, transported, or stored under unsuitable conditions [2]. There are four main types of aflatoxins in nature: B1, B2, G1, and G2. Aflatoxin B1 (AFB1), which is highly toxic, is recognized as a Group 1A carcinogen [3]. It is primarily associated with hepatocellular carcinoma [4], and recent studies have revealed that the bioactivation and potentially toxic effects of AFB1 also occur in extra‐hepatic tissues, such as the gastrointestinal tract [5].

Inflammatory response and oxidative stress may be observed in tissue that has been exposed to AFB1 [6]. Previous studies have found that the mechanism of AFB1 cytotoxicity is associated with the formation of reactive oxygen species, which cause cell integrity and DNA damage [7]. Therefore, activation of the immune system, induction of apoptosis, and cell proliferation cause intestinal barrier damage [8]. AFB1 exposure may lead to changes in the composition of the gut microbiota, resulting in a decrease in beneficial bacteria or an increase in pathogenic bacteria [9]. However, there are few studies on the effect of AFB1 on gut microbiota and oxidative stress pathways [5, 10].

Some chemical, physical, and biological methods have been developed for the elimination and inactivation of AFB1, as it poses a significant threat to human health [11]. The detoxification of AFB1 requires greater innovation and a higher rate of effectiveness considering damage to the organoleptic properties of food, loss of nutrients, low acceptability, and high cost [12]. Propolis, which is among the natural bioactive substances produced by honey bees, may be a promising method to detoxify AFB1 [13]. Moreover, studies have demonstrated that propolis prevents aflatoxin contamination in foods by inhibiting the growth of *Aspergillus spp*. fungi and the expression of genes involved in aflatoxin biosynthesis [14].

Propolis contains more than 300 polyphenols, monoterpenes, amino acids, steroids, phenolic aldehydes, and other inorganic compounds [15]. Studies have shown that the mechanism of these phenolic compounds emanates strong antioxidant activity by inhibiting free radicals [16]. Some studies have been conducted to investigate the protective effects of propolis against oxidative stress and its role in regulating the gut microbiota composition [17]. It was determined that propolis increased the activity of antioxidant enzymes, total antioxidant capacity, gut microbiota diversity, and richness in the oxidative stress model induced by dextran sulfate sodium [18]. In a similar study conducted with rats, it was found that propolis supplementation improved the gut microbiota composition and increased the expression of tight junction proteins [19].

Although the effects of propolis supplementation in modulating the intestinal microbiome and reducing oxidative stress have been investigated, the lack of studies on the prevention of gut microbiota dysbiosis and intestinal barrier damage in AFB1 toxicity remains a gap in the literature. In this study, the protective effect of propolis supplementation following AFB1 exposure was comprehensively investigated for the first time. This study primarily aimed to examine the effects of low‐dose AFB1 exposure on systemic oxidative stress, intestinal permeability, and gut microbiota composition. Secondly, it set out to assess the protective effects of propolis supplementation on AFB1‐induced biochemical, histological, and immunohistochemical changes.

## 2 Experimental Section

### 2.1 Preparation of AFB1 Content and Extraction of Propolis

The AFB1 (5 mg, ≥ 99.5%) (CAS No.: 1162‐65‐8, Cayman Chemical Company, Michigan, USA) was stored in its original packaging at 2–8°C prior to the commencement of the study. Dimethyl sulfoxide (DMSO, ≥ 99.5%) (CAS No.: 67‐68‐5, Sigma‐Aldrich Chemical Company, St. Louis, Missouri, USA) was selected as the solvent for AFB1 due to its minimal impact on animals [20]. The solution was prepared at a concentration of 5 mg/mL with a pH of 7.2.

Propolis used in this study was extracted from brown raw propolis (*Populus sp*.) collected from Samsun province, Türkiye. Propolis extracts were prepared according to the protocol described by Xue et al. [19]. The components of propolis extract determined by liquid chromatography–mass spectrometry (LC‐MS/MS) analysis are presented in Table S1.

### 2.2 Dosage Regimen

Previous studies have shown that administering AFB1 to rats at a daily dose of 25 µg/kg body weight for 4 weeks induces toxic effects. Due to differences in individual dietary intake, challenges in ensuring the homogenization of AFB1 levels in feed, and the need for dose control and reproducibility, oral gavage administration of AFB1 was preferred [9, 20]. In this study, the daily dose of AFB1 was determined as 25 µg/kg and administered to rats by oral gavage for 4 weeks. This dose is equivalent to 0.03–0.45 mg/kg (30–450 ppb) of AFB1 in food. Individuals living in developing countries have been reported to be exposed to AFB1 in this dose range [21].

Daily supplementation of 250 mg/kg propolis to rats increased the activities of antioxidant enzymes and reduced tissue damage [22]. In addition, propolis has demonstrated the ability to interact with the host microbiota when administered via oral gavage [23]. Based on previous studies, propolis was administered to rats by oral gavage at a dose of 250 mg/kg/day for 4 weeks [22]. Results of experimental animal studies have reported that the safe dose of propolis in humans is 70 mg/day [24]. The dose of propolis supplemented in this study was converted based on the equivalent dose relationship between rats and humans, resulting in approximately 8.1 mg/day.

### 2.3 Experimental Animals and Study Design

**FIGURE 1:**
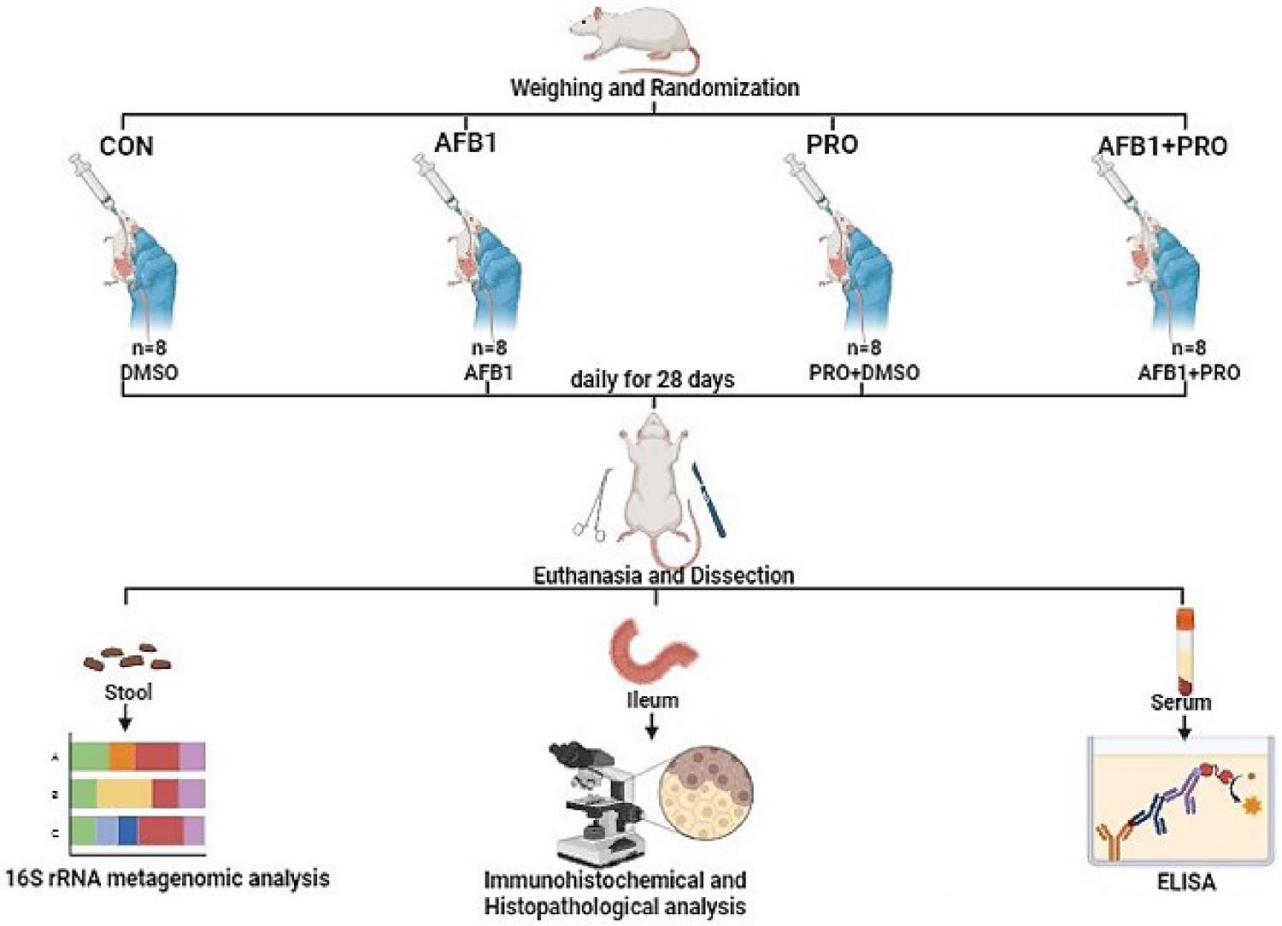
Study protocol. AFB1 indicates Aflatoxin B1‐exposed group; CON, control group; PRO, propolis‐supplemented group.

This study worked with 32 male Sprague‐Dawley albino rats aged 6–8 weeks (200–250 g). All procedures performed on animals were approved by the Local Ethics Committee for Animal Experiments, Erciyes University, Kayseri, Türkiye (Approval number: 22/234; Date: November 2, 2022). ARRIVE guidelines for animal welfare and handling, as well as the 3Rs (replacement, reduction, and refinement), were strictly applied [25]. Animals were separated randomly into cages, and the cages were randomly selected before any treatment was given. The animals were housed in individual polycarbonate cages with ad libitum access to a standard pellet diet (Table S2) and normal tap water. Environmental conditions were maintained at a constant temperature (22 ± 2°C) and humidity level (55% ± 10%) on a 12‐h light/dark cycle. An adaptation period of 1 week was applied to monitor the health status of the animals and to prevent stress. After the adaptation period, the animals were randomly divided into four groups. The study protocol is shown in Figure 1.

■ Control group (CON): DMSO diluted for 4 weeks (*n* = 8).
■ Aflatoxin B1‐exposed group (AFB1): 25 µg/kg AFB1 daily by oral gavage for 4 weeks (*n* = 8).
■ Propolis‐supplemented group (PRO): 250 mg/kg propolis daily by oral gavage for 4 weeks (*n* = 8).
■ Aflatoxin B1‐exposed and propolis‐supplemented group (AFB1+PRO): 25 µg/kg AFB1 and 250 mg/kg propolis by oral gavage for 4 weeks (*n* = 8).

### 2.4 Sample Collection and Biochemical Analyses

A combination of the injectable anesthetics xylazine and ketamine (10 and 80 mg/kg, respectively) was used for euthanasia. When reflexes, such as leg withdrawal and eyelids, were completely lost, maximum blood sampling was performed by cardiac puncture. The rats were then sacrificed by cervical dislocation. Blood samples were centrifuged to obtain serum (3000 rpm, 15 min). The ileum tissues of the animals were then dissected. Serum samples were stored at −80°C freezer for biochemical analyses. To examine systemic oxidative stress [26], serum levels of catalase (CAT) (Cat. No.: E0869Ra), glutathione (GSH) (Cat. No.: EA0113Ra), glutathione peroxidase (GSH‐PX) (Cat. No.: E1242Ra), malondialdehyde (MDA) (Cat. No.: E0156Ra), superoxide dismutase (SOD) (Cat. No.: E0168Ra), and total antioxidant capacity (T‐AOC) (Cat. No.: E1710Ra) were analyzed using commercially available rat ELISA kits (BT‐LAB, Shanghai, China) according to the manufacturer's protocol. Biochemical analysis results were recorded at 450 nm using an ELISA reader (Thermo Fisher Multiskan GO, Japan).

### 2.5 Histological Analyses

Ileum tissue obtained from sacrificed rats was placed in 10% formalin. After 72 h in the fixing solution, the tissues were washed in tap water for 1 h and then dehydrated by passing through a graded alcohol series (50%–100%). After the tissues were rendered transparent with xylene, they were embedded in paraffin and blocked. Serial sections of 5 µm were taken from the paraffin blocks and spread on slides. Paraffin was removed from the obtained sections by standard histological methods and diluted by passing through graded alcohol series. In order to see the general histological structure, the sections were stained with Hematoxylin & Eosin (H & E) staining and passed through increasing alcohol series and xylene, and the ileum morphology was examined under a microscope (Leica DM500, Wetzlar, Germany).

### 2.6 Immunohistochemical Analyses

For immunohistochemical evaluation, 5 µm serial sections were taken from paraffin blocks and spread on polylysine‐coated slides. The prepared slides were de‐paraffinized with xylol using standard histological methods and diluted with graded alcohol series. After optimization of primary Claudin‐1 (Cat. No.: 71–7800), occludin (Cat. No.: 71–1500), and Zonulin‐1 (Cat. No.: 61–7300) antibodies (Thermo Fisher, Cheshire, UK), immunohistochemical staining was performed by applying standard protocol in accordance with the manufacturer's recommendations. Photographs were taken from the prepared preparations with a Leica microscope at 10× magnification and the intensity of antibodies was measured in ImageJ program (Version 1.8.0_112).

### 2.7 Bacterial DNA Isolation and Sequencing of the 16S rRNA Gene

The feces of the rats were collected at the end of the fourth week through metabolic cages and each sample was placed in 2 mL sterile tubes. Samples were stored at −80°C freezer for analysis. Bacterial genomic DNA was isolated from rat fecal samples (100 mg) using a DNA extraction kit (Cat No.: E3551) (Gene Matrix, EURx, Gdansk, Poland) according to the manufacturer's recommendations. 16S ribosomal RNA (16S rRNA) Amplicon Metagenome sequencing method was used to evaluate the bacterial species found in the gut microbiota. In the library preparation steps before sequencing, 16S rRNA V3–V4 regions were analyzed with barcoded PCR primers and purified. After the library preparation steps were completed, the quality of the libraries was checked by fluorometric methods. Illumina Novaseq 6000 (San Diego, California, USA) next‐generation sequencing platform was used for sequencing.

### 2.8 Taxonomic Classification and Data Analysis

The quality of unprocessed raw reads was assessed using FastQC software (Novogene Company, Cambridge, UK). After adapter trimming was performed on the raw reads, quality filtering was performed with FastQC. The QIIME2 dada2 tool (version 3.2.0) and R software (version 4.3.0) were used for taxonomic assignment and identification of operational taxonomic units (OTUs).

### 2.9 Statistical Analysis

The number of rats to be used in the study and group allocation was determined by power analysis (*α* = 0.05 and *β* = 95%). G*Power software (Version 3.1.9.7, Kiel, Germany) was used to calculate the sample size. Accordingly, a total of 32 rats were used, with 8 rats in each group [26]. Data were analyzed using SPSS software (Version 26, IBM, Chicago, Illinois, USA). Variables assessed for normality using the Kolmogorov–Smirnov test are presented as the mean ± standard deviation (SD). In addition, the groups were compared using a one‐way ANOVA test, and a Tukey post‐hoc test was applied to variables with statistically significant differences. Similarly, OTU abundance comparisons in the metagenomic study groups were performed using the Kruskal–Wallis test. In statistical evaluations, *p* < 0.05 was considered statistically significant.

## 3 Results

### 3.1 Effects of AFB1 and Propolis on Serum Biochemical Parameters

**FIGURE 2:**
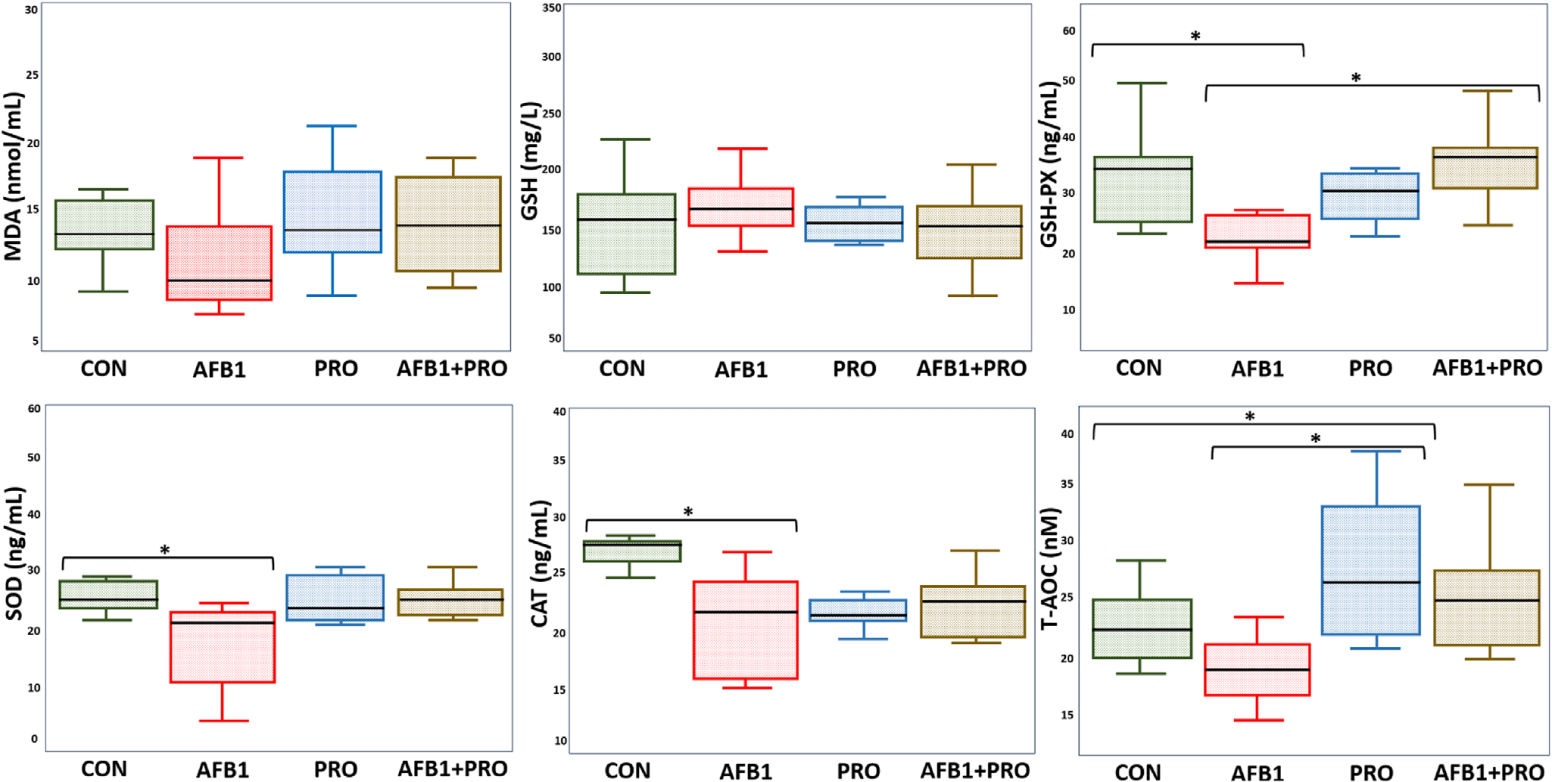
Evaluation of various oxidative stress parameters in serum of experimental groups. Study data were obtained from a single experiment. Three serum samples were taken from each of the eight animals in each group (*n* = 4) and a total of 96 (4 × 8 × 3) serum samples were examined. Data are expressed as the mean ± SD (*n* = 8 per group). One‐way ANOVA and then Tukey post‐hoc test were used to analyze the data. * means *p* < 0.05. CAT indicates catalase; GSH, glutathione; GSH‐PX, glutathione peroxidase; MDA, malondialdehyde; SOD, superoxide dismutase; T‐AOC, total antioxidant capacity.

The MDA level, which is an indicator of lipid peroxidation, and antioxidant enzyme levels such as GSH‐PX, SOD, CAT, T‐AOC, and GSH were measured in the serum of rats after AFB1 exposure and propolis supplementation. The GSH and MDA levels of rats did not appear statistically different between the groups (*p* > 0.05). GSH‐PX levels were significantly decreased in the AFB1 group compared to the CON group (*p* = 0.030), while no significant change was observed in the propolis‐supplemented groups (PRO and AFB1+PRO) (*p* > 0.05). When SOD and CAT levels were analyzed, the AFB1 group showed the lowest enzyme activity (*p* = 0.039 and *p* = 0.059, respectively). CAT levels tended to decrease in the propolis‐supplemented groups compared to the CON group, but this decrease was not significant (*p* > 0.05). The T‐AOC level in the PRO group was significantly higher than in the CON (*p* = 0.027) and AFB1 groups (*p* = 0.043). The T‐AOC level in the AFB1+PRO group was similar to that of the other groups (*p* > 0.05) (Figure 2).

### 3.2 Effects of AFB1 and Propolis on Histopathological Changes

**FIGURE 3:**
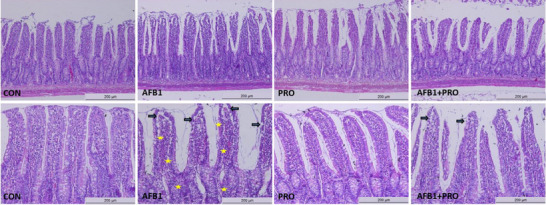
H & E staining results of ileum sections (*n* = 8 per group). Study data were obtained from a single experiment. Three sections were taken from each of the eight animals in each group (*n* = 4) and a total of 96 (4 × 8 × 3) sections were examined. Scale bar: 200 µm, Objective: 10×. Arrows: intestinal villus ruptures; yellow signs: inflammatory cells.

The histological structure of intestinal epithelial cells in rats was examined using H & E staining. The CON group showed a normal histological appearance, and villi were found to be structurally intact. Disorders in the intestinal villi, increased rupture, and inflammatory cell infiltration were observed in the AFB1 group. When the histological structure of AFB1+PRO group was examined, it was determined that propolis improved the intestinal morphological damage (Figure 3).

### 3.3 Effects of AFB1 and Propolis on Immunohistochemical Changes

**FIGURE 4:**
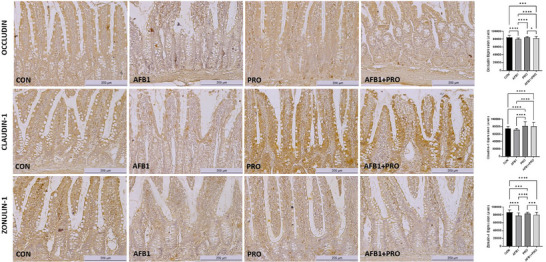
Immunohistochemical staining of occludin, claudin‐1, and zonulin‐1 proteins in the ileum of the experimental groups and immunoreactivities of these proteins. Scale bar: 200 µm, Objective: 10×. Study data were obtained from a single experiment. Three sections were taken from each of the eight animals in each group (*n* = 4) and a total of 96 (4 × 8 × 3) sections were examined. Data are expressed as the mean ± SD (*n* = 8 per group). One‐way ANOVA test followed by Tukey post‐hoc test was used to analyze the data. *p < 0.05, **p < 0.01, ***p < 0.001, ****p < 0.0001.

To investigate the effects of AFB1 exposure and propolis supplementation on the tight junction structure of intestinal epithelial cells in rats, occludin, Claudin‐1, and Zonulin‐1 expression in ileum tissue was evaluated through immunohistochemical staining. Occludin, Claudin‐1, and Zonulin‐1 immunoreactivity were found to be prominent in the lamina epithelialis and lamina propria (Figure 4). The expression of occludin was lower in the AFB1 group compared to the AFB1+PRO group (*p* < 0.0001). Claudin‐1 expression was significantly increased in the PRO and AFB1+PRO groups, with expression levels higher in these groups compared to the AFB1 group (*p* < 0.0001). The expression of Zonulin‐1 decreased in the experimental groups (*p* < 0.001). Additionally, the expression levels of all tight junction proteins were lower in the AFB1 group, while the expression levels were higher in the PRO group compared to the AFB1 group (*p* < 0.0001).

### 3.4 Effects of AFB1 and Propolis on Gut Microbiota

**FIGURE 5:**
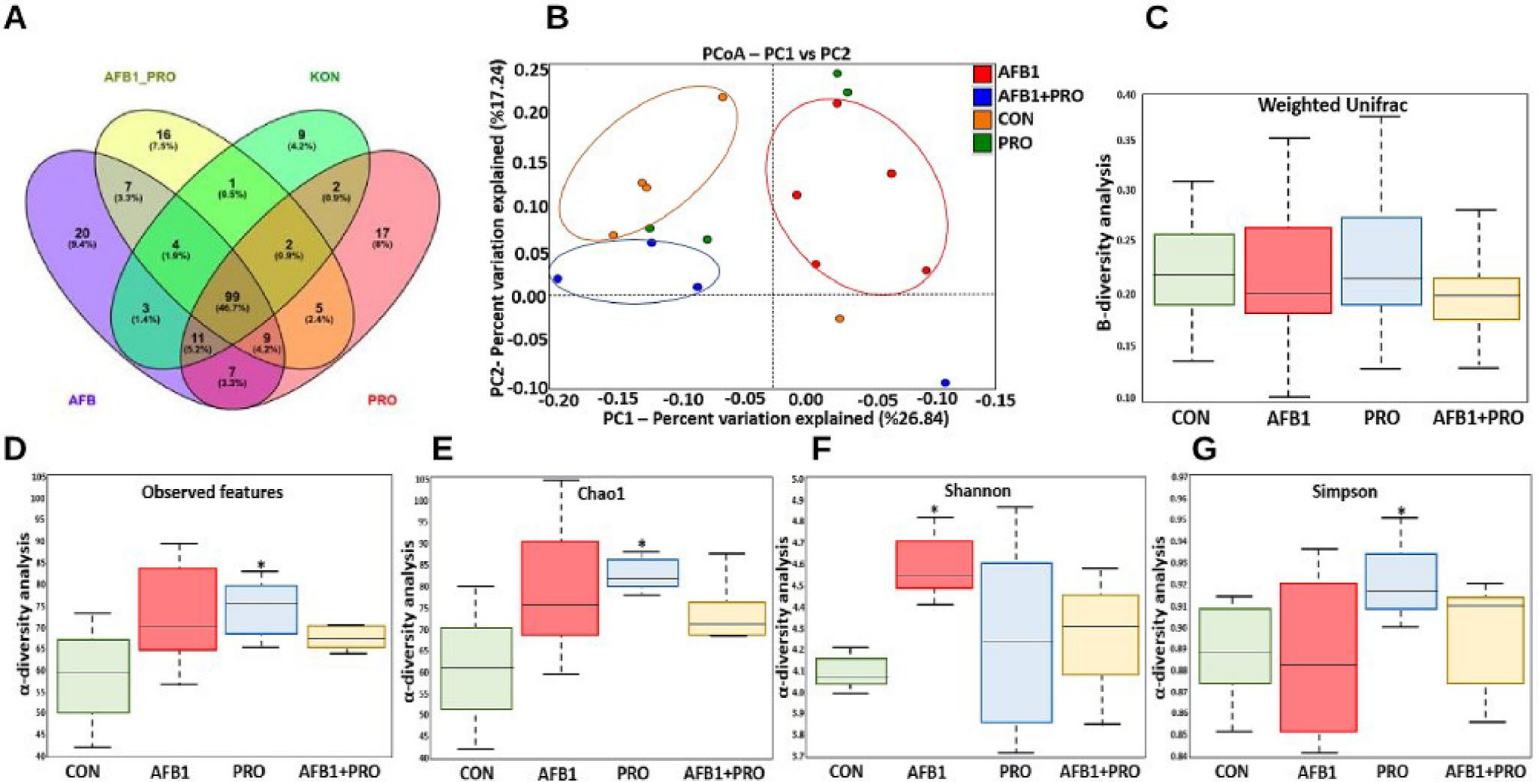
(A) Venn diagram of OTUs in experimental groups. (B) Principal coordinate analysis (PCoA) of gut microbiota from experimental groups based on the Bray–Curtis dissimilarity index. (C) Beta diversity weighted UniFrac distance matrix. (D–G) Observed features, Chao 1, Shannon and Simpson indices of *α*‐diversity analysis. The study data were obtained from a single experiment. Two fecal samples were taken from each of the eight animals in each group (*n* = 4) and a total of 64 (4 × 8 × 2) fecal samples were examined. Data are expressed as the mean ± SD (*n* = 8 per group in A–G). Data were analyzed using the Kruskal–Wallis test, *means *p* < 0.05 versus control group.

The effects of AFB1 exposure and propolis supplementation on the gut microbiota of rats were analyzed using the 16S rRNA gene sequencing method. A Venn diagram was used to examine the microbial community structure in the experimental groups, and the common and unique OTUs were explored. Most OTUs were shared among the four experimental groups (46.7%). The Venn diagram revealed that the AFB1 group contained more specific OTUs (Figure 5A). Principal coordinate analysis (PCoA), based on the Bray–Curtis dissimilarity index, was used to measure beta diversity between the experimental groups. It was observed that the fecal bacterial communities in the rats from the CON and AFB1+PRO groups were more similar to each other than those in the AFB1 and PRO groups. Moreover, these groups were clearly separated into distinct clusters, as shown by PC2. It seems quite clear, therefore, that AFB1 exposure and propolis supplementation altered the gut microbiota community structure in rats (Figure 5B). According to the weighted UniFrac distances, no significant difference was observed between the groups in terms of species diversity (Figure 5C). However, α‐diversity analysis (Observed features, Chao1, Shannon, and Simpson) revealed that bacterial community diversity in the PRO group was higher than in the other experimental groups (Figure 5D–G).

**FIGURE 6:**
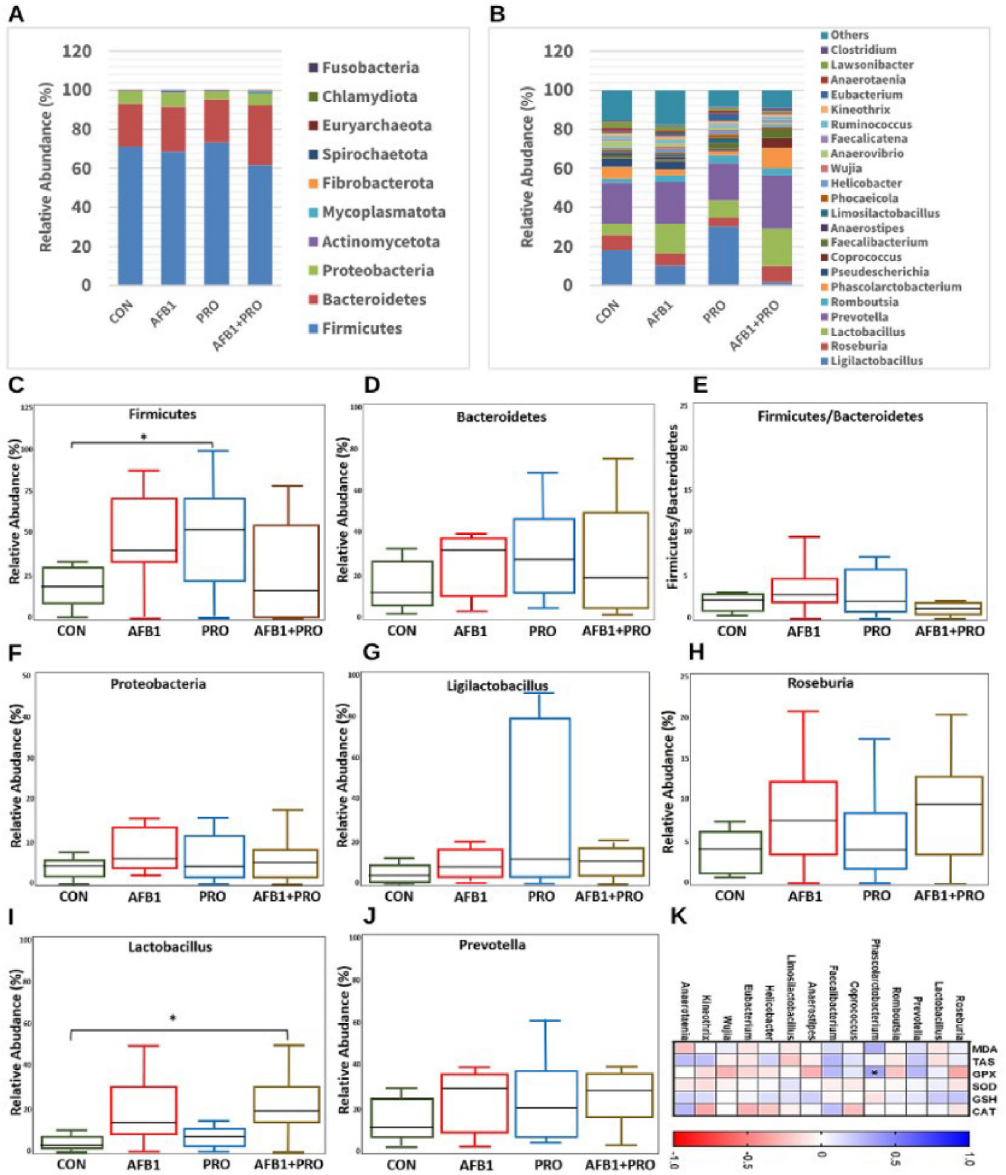
(A) Phylum level composition of gut microbiota. (B) Genus level composition of gut microbiota. (C, D, and F) Difference in gut microbiota (*Firmicutes, Bacteroidetes*, *and Proteobacteria*) composition at the phylum level. (E) *Firmicutes/Bacteroidetes* ratio. (G–J) Difference in the composition of gut microbiota (*Ligilactobacillus*, *Roseburia*, *Lactobacillus*, and *Prevotella*) at the genus level. (K) Heat map of the relationship between gut microbiota and oxidative stress parameters. The study data were obtained from a single experiment. Two fecal samples were taken from each of the eight animals in each group (*n* = 4) and a total of 64 (4 × 8 × 2) fecal samples were examined. Data are expressed as the mean ± SD (*n* = 8 per group in A–K). Data were analyzed using the Kruskal–Wallis test and Pearson's correlation test, *means *p* < 0.05 versus control group.

Relative abundances at the phylum and genus levels were examined to further elucidate the changes in gut microbial composition caused by AFB1 exposure and propolis supplementation. A total of 10 bacterial phyla were detected in the fecal samples of rats. Among these phyla, *Firmicutes, Bacteroidetes*, and *Proteobacteria* were the most abundant phyla in all experimental groups (Figure 6A). As shown in Figure 6C, the abundance of *Firmicutes* was significantly higher in the PRO group than in the CON group, accounting for approximately 73.51% and 71.01% of the bacterial abundance, respectively. The abundances of *Bacteroidetes* and *Proteobacteria* were found to be similar across the experimental groups (Figure 6D,F). At the same time, the *Firmicutes/Bacteroidetes* ratio (F/B) tended to increase in the AFB1 group, but no significant difference was detected between the groups (Figure 6E). A total of 22 species were screened at the genus level. Although *Ligilactobacillus, Roseburia, Lactobacillus*, and *Prevotella* were present in different proportions in the experimental groups, these genera constituted the majority (Figure 6B). When the experimental groups were compared in terms of relative abundance at the genus level, the *Lactobacillus* abundance in the AFB1+PRO group was significantly higher than in the CON group (*p* = 0.049) (Figure 6I). Although not statistically significant, *Phascolarctobacterium* abundance was higher in the AFB1+PRO group than in the AFB1 group (not shown in the figure) (*p* > 0.05). In addition, as shown in Figure 6K, a positive correlation was found between *Phascolarctobacterium* and GSH‐PX levels (Pearson's *r* = 0.355, *p* = 0.048).

**FIGURE 7:**
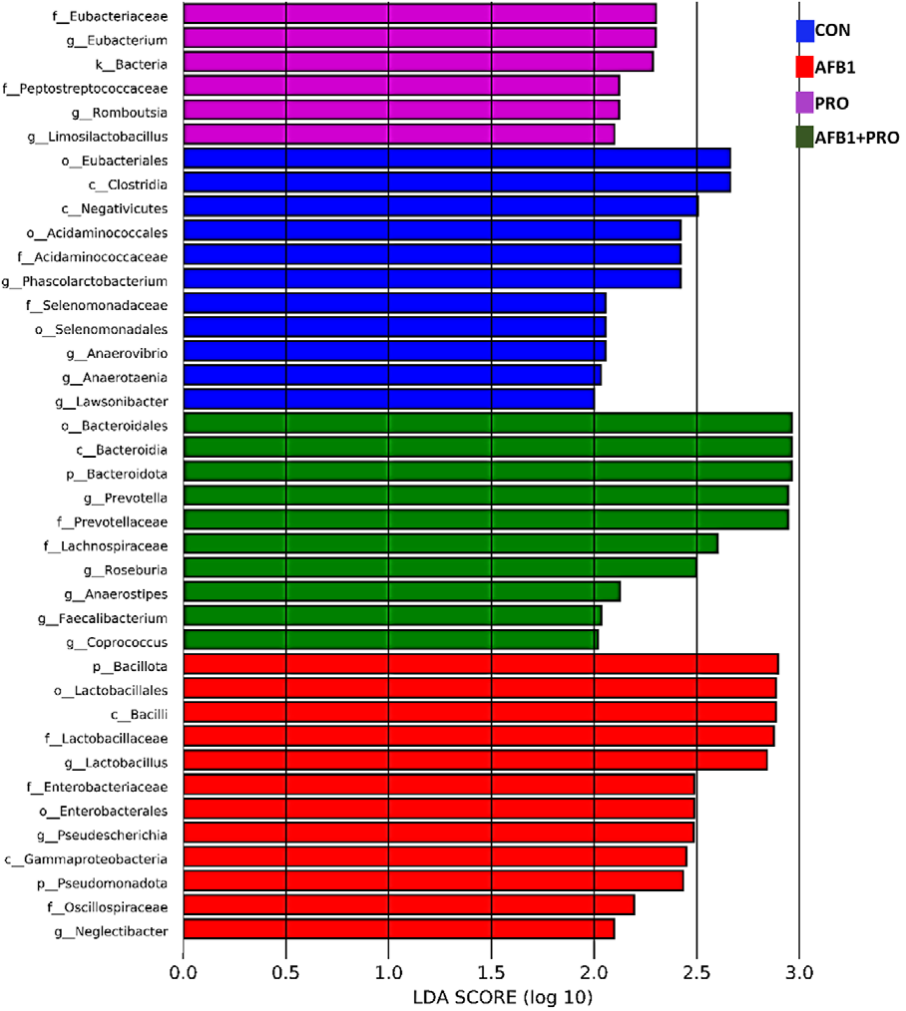
Comparison of LEfSe analysis results of gut microbiota in experimental groups (*n* = 8 per group). Each bar represents the log 10 effect size (LDA score) for a specific taxon. A longer bar represents a higher LDA score. The study data were obtained from a single experiment. Two fecal samples were taken from each of the eight animals in each group (*n* = 4) and a total of 64 (4 × 8 × 2) fecal samples were examined.

LEfSe analysis was performed to identify specific changes in bacterial taxonomy after propolis and AFB1 treatment. As shown in Figure 7, that the dominant bacterial phylum in the control group was Clostridia. Several genera were significantly increased in PRO, AFB1, and AFB1+PRO groups (LDA score > 2, *p* < 0.05). These are *Eubacterium, Romboutsia, Limosylalactobacillus; Lactobacillus, Pseudescherichia, Neglectibacter; Roseburia, Anaerostipes, Faecalibacterium*, and *Coprococcus* bacterial genera, respectively.

## 4 Discussion

AFB1 is found in various agricultural products, such as wheat, maize, and nuts, and may cause mycotoxicosis in humans and animals when contaminated foods and feeds are consumed [27]. This toxin has been reported to have hepatotoxic [28], neurotoxic [29], mutagenic [30], teratogenic [31], immunogenic [32], genotoxic [33], and carcinogenic [34] effects. Due to the widespread toxic effects of AFB1, research on this subject has accelerated in the recent past. Biological detoxification methods are being developed to reduce and prevent side effects caused by aflatoxicosis. For this purpose, probiotics [35] and antioxidant compounds [36] have come into popular usage as detoxification agents. Although previous studies have shown that antioxidants reduce the toxicity of AFB1, the lack of sufficient studies on the prevention of metabolic changes in intestinal tissue highlights a clear gap in the literature. In addition, no study has investigated the effects of propolis, which contains many antioxidant compounds, on AFB1‐induced oxidative stress, intestinal tissue damage, and gut microbiota composition were found. In this study, for the first time, the protective effect of propolis supplementation in response to low‐dose AFB1 exposure was comprehensively evaluated.

AFB1 is known to damage tissue through oxidative stress by increasing lipid peroxidation and inducing the formation of reactive oxygen species [37]. SOD, CAT, MDA, GSH, GSH‐Px, and T‐AOC biomarkers are commonly used to determine the oxidative stress caused by AFB1 in tissue [38]. In this study, serum oxidative stress biomarkers of rats were analyzed to assess the protective effects of propolis supplementation in AFB1‐exposed rats. CAT, GSH, GSH‐Px, and SOD are present in high amounts in mammalian cells and play important roles in antioxidant defense mechanisms [39]. These antioxidant markers help reduce oxidative stress by scavenging reactive oxygen species [40]. Previous studies have shown that AFB1‐induced dysbiosis associated with oxidative stress is linked to decreased T‐AOC levels, CAT, SOD, and GSH‐Px activities and increased MDA levels [41]. In this study, serum T‐AOC levels and SOD, CAT, and GSH‐Px activities were significantly decreased in AFB1‐exposed rats (Figure 2). These findings suggest that AFB1 impairs cellular defense mechanisms by reducing antioxidant capacity in the serum, leading to increased oxidative stress. The decreased activity of antioxidant enzymes may make cells more susceptible to the toxic effects of AFB1, resulting in further oxidative damage at both the cellular and tissue levels [42]. At the same time, no significant difference was observed in MDA levels between the groups. This may be due to the fact that the doses of AFB1 and propolis supplementation did not cause significant changes in MDA levels. The low levels of T‐AOC and antioxidant enzyme activities may indicate that cellular defense mechanisms are compromised after AFB1 exposure. It is possible that decreased antioxidant capacity and impaired defense mechanisms delay the detoxification of AFB1 metabolites and lead to the formation of reactive oxygen species [43]. This may result in increased oxidative tissue damage and impairment of metabolic functions [44].

Recent studies have shown that antioxidant compounds can reduce AFB1‐induced oxidative stress [45]. In this study, Anatolian propolis, which contains phytochemicals such as resveratrol, gallic acid, cyanidin, chlorogenic acid, catechin, vanillic acid, hesperidin, ferulic acid, quercetin, apigenin, and chrysin, was used. It is common knowledge that propolis reduces inflammation and oxidative stress by regulating the nuclear factor‐kappa B pathway due to the antioxidant compounds it contains [46]. This research noted that serum T‐AOC levels and GSH‐PX activity were found to be higher in propolis‐supplemented groups compared to AFB1 group (Figure 2). Additionally, MDA, GSH, SOD, and CAT levels in propolis‐supplemented rats were similar to those in the CON group. The improvement in antioxidant status in these experimental groups may be attributed to the antioxidant and anti‐inflammatory activities of propolis [47]. However, there is a limited number of studies on propolis supplementation in aflatoxicosis. Yilmaz et al. suggested that propolis supplementation might be a natural agent for preventing oxidative stress and hepatotoxicity in AFB1‐exposed rats [26]. Consequently, propolis supplementation may be a useful method for improving impaired detoxification metabolism and preventing tissue damage by reducing oxidative stress [48].

The intestinal barrier consists of epithelial cells, the mucus layer, and tight junctions that protect the intestine against infections and pathogens. Tight junctions play a major role in the regulation of intestinal barrier permeability [49]. Occludin, Claudin‐1, and Zonulin‐1 are tight junction proteins. Exposure to oxidative stress factors such as mycotoxins may decrease the expression of these proteins and cause disruption of the intestinal mucus barrier [50]. In various in vivo models, it has been shown that AFB1 exposure disrupts the intestinal barrier structure and decreases the expression of tight junction proteins [51]. The results of this research demonstrate that in AFB1‐exposed rats damaged the intestinal barrier structure by causing disorder, rupture of intestinal villi (Figure 3), and decreased the expression levels of occludin, Claudin‐1, and Zonulin‐1 (Figure 4). Furthermore, propolis supplementation ameliorated the histomorphological damage caused by AFB1 exposure, protected against intestinal barrier disruption, and decreased intestinal permeability by increasing the expression of tight junction proteins. Thus, propolis supplementation is thought to contribute to the maintenance of intestinal health by protecting the intestinal barrier against harmful stimuli.

Gut microbiota consists of a complex and dynamic ecosystem involved in functions such as the protection of intestinal epithelium, regulation of the immune system, and nutrient and drug metabolism [52]. AFB1 is known to affect bacterial species diversity and richness [53]. Peng et al. reported that AFB1 exposure increased Chao1 and ACE indices, which show species richness, and Shannon and Simpson indices, which show species evenness [54]. Consistent with the literature, Chao1 and Shannon index values were significantly increased in AFB1‐exposed rats (Figure 5E,F). When the rats were supplemented with propolis, bacterial species diversity and richness were similar to the CON group. At the same time, the AFB1 group was found to contain more specific OTUs (Figure 5A). In a previous study, AFB1 exposure resulted in a decrease in the number of OTUs compared to the control group [55]. It seems clear that AFB1 exposure can affect the growth of some specific species and the number of OTUs in the gut microbiota. Wang et al. revealed that AFB1 changed the bacterial community structure and differentiated from the control group [9]. Similarly, it was shown by the Bray–Curtis dissimilarity index that the fecal bacterial communities of rats in the CON and AFB1+PRO groups were more similar to each other than the rats in the AFB1 group (Figure 5B). According to the results obtained, it can be said that propolis supplementation is effective on AFB1‐induced altered bacterial community structure.

Previous studies have also shown that AFB1 changes the composition of the gut microbiota, reducing the number of beneficial bacteria [20a]. In this study, phylum and genus level changes were examined in fecal samples of AFB1‐exposed rats using amplicon sequencing. *Firmicutes* and *Bacteroidetes* were found to be the two most dominant phyla in the gut microbiota of all experimental groups. The F/B ratio is associated with inflammatory marker levels and the pathological condition of intestinal metabolic homeostasis [56]. It has also been suggested that AFB1 causes inflammation, oxidative stress, and obesity by increasing the F/B ratio [57]. However, the protective effects of propolis on gut microbiota in AFB1‐exposed rats remain unclear. The results of our study support the findings that propolis alleviates oxidative stress by restoring the F/B ratio increased by AFB1 exposure [58]. In addition, several remarkable bacterial genera were detected in all experimental groups as follows: *Lactobacillus, Roseburia*, and *Phascolarctobacterium*. Lactic acid bacteria have been reported to bind to AFB1 in some studies [59]. *Lactobacillus* strains play a role in detoxification by preventing AFB1 binding to the cell wall [60]. *Lactobacillus*, which is normally present in the gut microbiota, provides inhibition of pathogenic bacteria colonized in the intestine [61] and supports the repair of intestinal mucosa by exhibiting anti‐inflammatory properties [62]. *Roseburia* is among the butyrate‐producing bacteria and is usually found in the colon [63]. *Roseburia* species, whose relative abundance increases as a result of consumption of foods with prebiotic properties, have been shown to contribute to the recovery from diseases such as inflammatory bowel disease, type 2 diabetes, and atherosclerosis, thanks to some metabolites they produce [63, 64]. A positive correlation was found between the genus *Phascolarctobacterium*, which metabolizes prebiotics to produce SCFAs such as acetate and propionate, and serum GSH‐PX activity. It has been suggested that *Phascolarctobacterium* bacteria prevent oxidative stress by reducing the production of inflammatory cytokines [65]. Moreover, in this research, *Phascolarctobacterium* abundance was higher in the AFB1+PRO group than in the AFB1 group. Propolis supplementation prevented the decrease in *Lactobacillus, Roseburia*, and *Phascolarctobacterium* caused by AFB1 exposure. These results indicate that propolis supplementation affected gut microbiota composition and may be effective in eliminating AFB1‐induced dysbiosis.

LEfSe analysis was performed to identify specific changes in bacterial taxonomy after propolis and AFB1 treatment. By screening at the genus level, genera that deserve attention were identified in the experimental groups. In the AFB1 group, the recently identified gram‐negative genus *Pseudescherichia* [66], which has been found to cause sepsis, was significantly increased. Thus, it can be concluded that AFB1 exposure increases the pathogenic bacterial population. At the same time, in the AFB1+PRO group, the abundance of *Anaerostipes* [67] and *Coprococcus* [68], which produce SCFAs and are used in the treatment of inflammatory bowel diseases by reducing oxidative stress, was clearly increased. Furthermore, it was established that the prebiotic phenolic compounds in propolis [69] may provide a substrate for *Anaerostipes* and *Coprococcus* bacteria and promote their growth.

The comprehensive investigation of the effect of propolis supplementation on dysbiosis (biochemical, histological, immunohistochemical, and amplicon sequencing) in AFB1‐exposed rats is one of the strengths of this study. On the other hand, to better understand the effects of propolis supplementation on AFB1‐induced dysbiosis, it is thought that a longer study period and different propolis doses may be beneficial in future research.

The present study demonstrated for the first time the protective effects of propolis supplementation against dysbiosis induced by low‐dose AFB1 exposure. Propolis supplementation is thought to contribute to the modulation of gut microbiota by alleviating oxidative stress, ameliorating intestinal barrier damage, and reducing intestinal permeability in AFB1‐exposed rats. In addition, AFB1 exposure has been shown to specifically increase some pathogenic bacterial genera, whereas propolis supplementation leads to an increase in beneficial bacterial genera. However, the potential mechanisms of propolis in AFB1 detoxification have yet to be adequately determined. Therefore, the AFB1 detoxifying effect of propolis, when used as a biological agent, should be investigated more extensively.

## Conflicts of Interest

The authors declare no conflicts of interest.

## Supporting information



Supporting Information

Supporting Information

## Data Availability

Data available on request from the authors.
