# Supplementary material for: Protective Effects of Propolis Supplementation on Aflatoxin B1‐Induced Oxidative Stress, Antioxidant Status, Intestinal Barrier Damage, and Gut Microbiota in Rats
Source: Mol Nutr Food Res. 2025 Mar 30;69(10):e70052. doi: 10.1002/mnfr.70052 (PMC12087736; doi:10.1002/mnfr.70052)
Supplement: Supplementary file 1 — Supporting Information [file MNFR-69-e70052-s001.docx]

**Supplementary table S1**

LC/MS/MS analysis of phenolic compounds in propolis

| **Quantitative Analysis Summary Report** | | | | | |
| --- | --- | --- | --- | --- | --- |
| **Batch Data Path** | D:\MassHunter\Data\2023\06-Haziran\Phenolic_Compounds\QuantResults\Phenolic_Compounds_Propolis.batch. | | | | |
| **Analysis Time** | 6/16/2023 5:23 PM | **Analyst Name** | LCMS\admin |  |  |
| **Report Time** | 6/16/2023 5:24 PM | **Reporter Name** | LCMS\admin |  |  |
| **Last Calib Update** | 6/16/2023 5:23 PM | **Batch State** | Processed |  |  |
| **Quant Batch Version** | B.07.01 | **Quant Report Version** | B.07.01 |  |  |
| **Sequence Table** |  |  |  |  |  |
| **Data File** | **Acq Method File** | **Sample Name** | **Sample Type** | **Position** | **Volume** |
| SK_Propolis-Num.d | Phenolic_Compounds.m | SK_Propolis-Num | Sample | P1-B5 | -1.00 |
| **Quantitation Results** |  |  |  |  |  |
| **Data File** | **Compound** | **Sample Type** | **Response** | **Final Conc** | **Unit** |
| SK_Propolis-Num.d | Quinic Acid | Sample | 7080 | 2683.9697 | ng/mL |
| SK_Propolis-Num.d | Fumaric Acid | Sample | 4028 | 2389.1864 | ng/mL |
| SK_Propolis-Num.d | Gallic Acid | Sample | 4919 | 147.9120 | ng/mL |
| SK_Propolis-Num.d | Pyrogallol | Sample | 5 | 0.0000 | ng/mL |
| SK_Propolis-Num.d | Keracyanin Chloride | Sample | 6171 | 1845.1999 | ng/mL |
| SK_Propolis-Num.d | Cyanidin-3-o-glucoside | Sample | 18030 | 1819.2552 | ng/mL |
| SK_Propolis-Num.d | Chlorogenic Acid | Sample | 45995 | 1718.2447 | ng/mL |
| SK_Propolis-Num.d | Catechin | Sample | 542 | 17.1751 | ng/mL |
| SK_Propolis-Num.d | Peonidin-3-o-glucoside | Sample | 741 | 148.2484 | ng/mL |
| SK_Propolis-Num.d | 4-OH-Benzoic Acid | Sample | 40668 | 2686.7663 | ng/mL |
| SK_Propolis-Num.d | Epicatechin | Sample | 145 | 0.0000 | ng/mL |
| SK_Propolis-Num.d | Epigallocatechin Gallate | Sample | 1 | 0.0000 | ng/mL |
| SK_Propolis-Num.d | Caffeic Acid | Sample | 2521239 | 49483.7460 | ng/mL |
| SK_Propolis-Num.d | Vanillic Acid | Sample | 1471 | 3004.1509 | ng/mL |
| SK_Propolis-Num.d | Syringic Acid | Sample | 11 | 0.0000 | ng/mL |
| SK_Propolis-Num.d | Vitexin | Sample | 8243 | 44.1778 | ng/mL |
| SK_Propolis-Num.d | Naringin | Sample | 74 | 0.0000 | ng/mL |
| SK_Propolis-Num.d | Ellagic Acid | Sample | 620 | 99.0671 | ng/mL |
| SK_Propolis-Num.d | Hesperidin | Sample | 3137 | 249.7517 | ng/mL |
| SK_Propolis-Num.d | p-Coumaric Acid | Sample | 1566896 | 31124.9127 | ng/mL |
| SK_Propolis-Num.d | Sinapic Acid | Sample | 24 | 0.0000 | ng/mL |
| SK_Propolis-Num.d | Taxifolin | Sample | 94743 | 2204.5354 | ng/mL |
| SK_Propolis-Num.d | Ferulic Acid | Sample | 179250 | 41525.3079 | ng/mL |
| SK_Propolis-Num.d | Rosmarinic Acid | Sample | 3708 | 920.3959 | ng/mL |
| SK_Propolis-Num.d | Vanillin | Sample | 2229 | 329.2110 | ng/mL |
| SK_Propolis-Num.d | Myricetin | Sample | 999 | 0.0000 | ng/mL |
| SK_Propolis-Num.d | Resveratrol | Sample | 0 | 0.0000 | ng/mL |
| SK_Propolis-Num.d | Luteolin | Sample | 292993 | 2731.7785 | ng/mL |
| SK_Propolis-Num.d | Quercetin | Sample | 479234 | 57654.7720 | ng/mL |
| SK_Propolis-Num.d | Apigenin | Sample | 68693 | 589.8101 | ng/mL |
| SK_Propolis-Num.d | Naringenin | Sample | 16604 | 234.4466 | ng/mL |
| SK_Propolis-Num.d | Isorhamnetin | Sample | 677420 | 4778.3925 | ng/mL |
| SK_Propolis-Num.d | Chrysin | Sample | 5384 | 88.2617 | ng/mL |
| SK_Propolis-Num.d | Galangin | Sample | 48417 | 5075.5507 | ng/mL |
| SK_Propolis-Num.d | Curcumin | Sample | 4 | 0.0000 | ng/mL |
| QuantReport_ESTD_Complete_B_06_00.xlsx | | | Printed at: 5:26 PM on: 6/16/2023 | | |
