# Supplementary material for: Protective Effects of Propolis Supplementation on Aflatoxin B1‐Induced Oxidative Stress, Antioxidant Status, Intestinal Barrier Damage, and Gut Microbiota in Rats
Source: Mol Nutr Food Res. 2025 Mar 30;69(10):e70052. doi: 10.1002/mnfr.70052 (PMC12087736; doi:10.1002/mnfr.70052)
Supplement: Supplementary file 2 — Supporting Information [file MNFR-69-e70052-s002.docx]

**Supplementary table S2**

Composition of standard pellet feed

| Nutrients | Units | Quantity |
| --- | --- | --- |
| Crude protein | % | 24 |
| Crude cellulose | % | 4 |
| Crude oil | % | 5.54 |
| Crude ash | % | 8.63 |
| Lizin | % | 1,45 |
| Methionine | % | 0.61 |
| Calcium | % | 1.14 |
| Phosphorus | % | 0.87 |
| Sodium | % | 0.76 |
| Iodine | mg/kg | 1.3 |
| Cobalt | mg/kg | 0.15 |
| Copper | mg/kg | 16 |
| Manganese | mg/kg | 155 |
| Zinc | mg/kg | 130 |
| Selenium | mg/kg | 0.35 |
| Vitamin A | IU/kg | 36000 |
| Vitamin D | IU/kg | 6500 |
